# Elderhood, Agency and Existential Vulnerability

**DOI:** 10.1093/geroni/igab046.1917

**Published:** 2021-12-17

**Authors:** H E Laceulle

**Affiliations:** University of Humanistic Studies, Utrecht, Netherlands, Utrecht, Netherlands

## Abstract

Popular conceptualizations of elderhood often use a spiritually inspired language of personal growth and wisdom. These conceptualizations are rightly critical of the language of activity and productivity that abounds in dominant successful aging discourses. Instead, the emphasis is placed on embracing our diminishing strength and increasing dependence with an attitude of resignation and gracious acceptance. Problematically, however, this can reinforce the ageist cultural assumption that old age lacks agency. If the emerging discourse about elderhood is truly to serve as a more inspiring cultural image of late life, it requires a reconceptualization of agency in the face of existential vulnerabilities. This paper aims to present a possible philosophical outlook for such a reconceptualization. It will draw on sources from feminist philosophy to argue how confrontations with vulnerability need not be an obstacle, but rather inspire alternative conceptualizations of agency that are a welcome addition to gerontological thinking.

